# 이행이론을 기반으로 한 폐경이행모형

**DOI:** 10.4069/kjwhn.2022.08.16

**Published:** 2022-09-30

**Authors:** Jisoon Kim, Sukhee Ahn

**Affiliations:** 1 Department of Nursing, College of Health and Welfare, Woosong University, Daejeon, Korea; 1 우송대학교 보건복지대학 간호학과; 2 College of Nursing, Chungnam National University, Daejeon, Korea; 2 충남대학교 간호대학

**Keywords:** Middle-aged, Psychological adaptation, Psychological resilience, Quality of life, Social support, 중년, 적응, 회복탄력성, 삶의 질, 사회적 지지

## Abstract

**Purpose:**

The purpose of this study was to construct a hypothetical model based on Meleis and colleagues' Transition Theory and a literature review to explain women’s menopausal transition, constructing a modified model considering previous studies and model fit and testing the effects between variables.

**Methods:**

With a correlational survey design, middle-aged Korean women aged 40 to 64 years who had experienced menopausal symptoms were recruited and filled out a self-administered study questionnaire. Measures included menopausal symptoms, resilience, social support, menopause management, menopause adaptation, and quality of life. The data were analyzed using SPSS 24.0 and AMOS 24.0.

**Results:**

The model fit indices were considered acceptable: *χ*^2^/degree of freedom=2.93, standardized root mean residual=.07, comparative fit index=.90, and parsimonious normed fit index=.73. All eight direct-effect paths—from menopausal symptoms to support and adaptation, from support to adaptation and resilience, from resilience to adaptation and management, from management to quality of life, and from adaptation to quality of life—were significant. The explanatory power of the menopause transition model was 63.6%.

**Conclusion:**

Women who experience menopausal symptoms may be able to maintain and improve their quality of life if menopause management and menopause adaptation are successful through resilience and social support. Future research is needed to confirm whether strengthening facilitation as a nursing intervention strategy may promote healthy response patterns.

## Introduction

여성은 폐경을 경험하면서 월경의 변화와 더불어 안면홍조와 같은 신체적 증상, 집중 장애와 같은 정신적 증상, 가족 내 역할 변화, 사회적 지지체계 변화, 대인관계 상실로 인한 사회•심리적 변화로 인해 불안정한 상태를 경험한다[1,2]. 이에 간호사는 폐경이행을 경험하는 여성을 대상으로 폐경증상 관리와 더불어 폐경 전•후 단계를 경험하며 통과하는 과정인 ‘이행’을 여성 생애주기의 정상적인 발달단계로 이해하고, 이들의 폐경기 적응과 삶의 질을 증진하려는 건강요구도에 부응할 수 있어야 한다.

임상적으로 폐경이행기의 시작은 생리가 불규칙해지거나 에스트로겐 결핍증상이 나타나기 시작하는 때로, 증상의 정도도 경미한 정도부터 심한 증상까지 개인적으로 다양하게 나타난다[3]. 폐경이행기 여성의 삶의 질에 미치는 영향요인으로는 폐경증상[1,4], 회복탄력성[5], 사회적 지지[6,7], 폐경관리[8], 폐경기 적응[9,10]을 보고하고 있다. 폐경이행기 여성이 심한 신체적, 정신적, 또는 심리적 증상을 경험하고 관리를 효율적으로 하지 못하면 삶의 질이 저하된다[4]. 그러나 이때 사회적 지지와 회복탄력성을 통해[6,11] 폐경기 증상을 효과적으로 관리하고 대처방안을 찾아 실천하며 폐경기 적응과 극복을 위한 다양한 노력을 통한 적응 과정을 거친다면[9,12,13], 삶의 질은 보다 향상될 수 있다[13-15]. 따라서 폐경이행을 단순히 폐경 자체가 아닌 폐경을 경험하면서 다양한 내•외적 자원을 활용하고 관리와 적응을 통해 삶의 질을 증진하는 과정으로 이해할 필요가 있다. 하지만 폐경이행기 여성의 삶을 이행의 관점으로 평가한 연구는 극히 제한적이다.

Schumacher와 Meleis [16]의 이행개념에 따르면, 이행은 인간이 그들의 삶이나 환경의 변화에 직면하여 장기간에 걸쳐 이루어지는 적응의 과정이다. 이행은 긍정적인 특성을 가지기 때문에, 이행을 마치고 나면 불안정했던 개인은 이전보다 더 안정된 상태에 도달하게 된다. 폐경기는 발달단계에 따른 건강한 이행과정으로, 대상자에게 이러한 이행이 일어날 때 주관적, 행동적, 대인관계 차원이 통합되어 건강상태에 영향을 미치기 때문에 간호사는 대상자들이 건강한 이행을 할 수 있는 상황을 생성하도록 지지해야 한다[16,17]. 폐경이행은 폐경 전 안정단계에서 폐경을 경험하고 폐경 후 안정단계를 통과하는 과정으로, 관리와 적응이 필요한 여성 생애주기의 정상적 발달단계이다. 폐경 속성이 이행의 과정에서 선행조건으로 시작하면 폐경이행 조건이 촉진요인으로 작용을 하고 폐경이행 반응 패턴에 영향을 주어 긍정적인 폐경이행 과정을 통과하게 된다[1]. 장기간의 적응과정과 불안정에서 안정으로의 긍정적 특성을 갖는 이행[16]에서 폐경증상은 종점이 되는 안정된 폐경으로 이행되는 과정 중 불안정한 증상으로서의 시점인 속성이 된다. 또한 폐경증상은 이행속성인 발달유형, 복잡한 패턴, 시간주기의 고유성의 속성을 지니고 있다[1].

회복탄력성과 사회적 지지는 이러한 폐경이행의 촉진 요인이 될 수 있다. 회복탄력성은 역경에 직면했을 때 원상태로 회복하는 대처능력으로서, 역동적으로 상호작용하는 과정을 통해 부정적 결과에서 긍정적인 결과를 가져온다[18,19]. 따라서 상황의 해석과 대처에 작용하는 신념과 태도인 회복탄력성은 자가관리[20], 적응[21]을 촉진할 것이다. 사회적 지지는 삶에 긍정적 영향을 미치는 사회•심리적 변수로, 부정적 정서 상태를 완화하고 스트레스 상황에서 대응과 적응을 돕는 매개이자 중개 역할을 한다[22]. 사회적 지지는 또한 회복탄력성에 영향을 미치는 요인이기에[19], 사회적 지지 역시 폐경이행의 촉진요인으로 기여할 것이다. 하지만 폐경증상에서 폐경관리, 폐경기 적응, 삶의 질에 이르기까지의 폐경이행 과정에서 회복탄력성과 사회적 지지를 내•외적 강화요인으로 보고 직∙간접효과[20]를 나타내는 촉진요인의 효과를 탐색한 연구는 적었다.

폐경이행 현상을 이행이론을 기반으로 탐색한 선행연구는 국내외 연구 2편이 보고되고 있다. Im과 Meleis [23]는 이행이론에서 유도한 양적, 질적 연구를 수행하고 폐경이행 특수상황이론을 개발하여 폐경이행 현상을 설명하는 이론적 근거를 제시하였다. 또 다른 연구[1]에서는 건강한 폐경이행 모형을 구축하고 이행조건과 이행반응패턴의 관계를 검증하였지만, 폐경이행 속성을 고려하지 못한 제한점이 있다. 폐경이행 속성은 폐경이행 과정을 이해하는 데 필요한 선행 조건이기 때문에, 이런 속성이 전제되어야 폐경이행에 대한 과정적 이해와 이행 반응을 연계하는 이론적 설명이 가능하다. 따라서 본 연구에서는 이행의 속성, 조건, 반응패턴으로 구성된 Meleis 등[17]의 이행이론에 근거하여 폐경이행 속성부터 이행 조건을 통한 이행 반응패턴을 설명할 수 있는 모형을 구성하고 검증하며, 이를 통해 폐경이행을 효과적으로 돕기 위한 간호전략 개발의 지침을 제공하고자 한다.

본 연구의 목적은 Meleis 등[17]의 이행이론을 기반으로 폐경이행기 여성에 관련된 폐경이행 모형을 구축하고, 모형의 구성개념인 폐경이행 속성, 폐경이행 조건, 폐경이행 반응패턴간의 직접•간접•총 효과를 검증하는 것이다. 구체적인 목적은 다음과 같다.

1)폐경이행기 여성의 폐경이행 과정을 설명하는 모형을 구축하고 실제 자료간 적합성을 검증한다.

2)폐경이행의 구성요소인 폐경이행 속성, 폐경이행 조건, 폐경이행 반응패턴 관계에서 직접 및 간접 효과의 경로를 규명한다.

**Figure 1.**
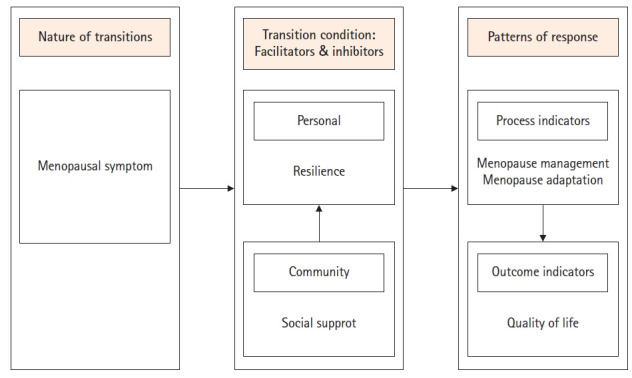
Conceptual framework of the menopausal transition in this study.

**Figure 2.**
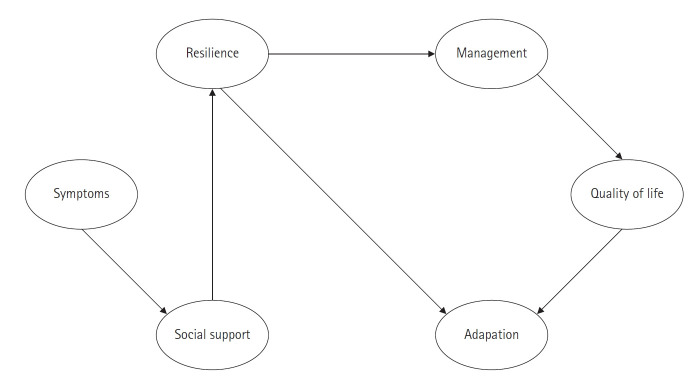
Initial hypothetical model.

Meleis 등[17]의 이행이론에서 이행은 변화에 직면할 때 안정된 상태들 사이의 기간으로 한 상황에서 다른 상황으로의 통과라고 정의된다. 이행이론은 이행의 속성, 이행의 조건(촉진요소 및 장애요소), 반응패턴, 간호중재의 4가지 주요개념으로 구성된다. 이행이론을 기반으로 한 폐경이행에 대한 개념적 기틀 및 가설모형은 Figures 1, 2와 같다.

폐경증상은 폐경이행 속성을 반영할 개념으로 대입할 수 있다. 이는 생애 주기인 중년기 여성의 내분비계 불균형으로 인한 신체변화를 경험하는 발달과정이기에, 이행 유형과 불안정한 변화에 대해 적응과 관리가 미흡할 경우 질병으로 진행될 건강•질병 이행 유형에 속한다. 신체변화 외에도 가족에서의 역할 변화, 사회적 지지체계의 변화, 대인관계 상실, 삶의 질 증진 및 저하에 대한 평가 등 다양하고 복잡한 패턴을 보인다. 폐경이행의 시작 신호인 월경의 불규칙성을 인지하고 이후의 불안정하고 혼돈된 시기를 거치게 되나 결정적인 사건인 폐경이라는 분명한 지점을 통과하게 되는 시간주기를 가지고 있다[1]. 따라서 개인마다 다양한 신체적, 심리적 폐경증상의 정도에 따라 폐경으로의 이행의 과정과 결과를 긍정적 또는 부정적으로 경험하게 되기 때문에[1,4], 폐경증상을 속성 개념으로 대입하였다.

폐경이행의 조건은 폐경이행의 속성이 폐경이행 반응패턴으로 통과하는 과정에 영향을 주는 요소이다. 선행연구 고찰을 통해 폐경이행의 조건에서 의미와 기대, 신념과 태도를 반영하는 개인적 조건으로 회복탄력성을, 폐경이행을 촉진하거나 방해할 수 있는 환경적 조건으로 사회적 지지를 설정하였다. 폐경이행을 건강하게 성취하려면 폐경이행 과정을 촉진하거나 방해하는 개인적, 환경적 조건을 밝히는 것이 필수요건이라 하였으므로[17] 폐경이행 조건을 반영할 개념으로 회복탄력성과 사회적 지지를 모형에 대입하였다.

폐경이행 반응패턴은 폐경이행 과정 동안 사회문화적 맥락뿐 아니라 정신내부의 구조와 과정을 반영하여 외부적으로 관찰 가능한 행동으로 표출되는 것이다[17]. 폐경이행을 건강하게 진행하도록 도우려면 폐경이행 반응패턴의 특징을 주기적으로 평가하기 위한 지표로 과정지표와 결과지표를 파악해야 한다. 과정지표로는 연결감, 상호작용, 자리매김과 상황에 처해있음, 자신감 발달 등의 개념을 반영하는 폐경기 적응과 대처를 반영하는 폐경관리를 설정하였다. 폐경기 적응은 폐경으로 인한 신체, 자아개념, 역할기능, 상호의존성 변화에 적응하는 여성의 삶의 질을 결정하는 중요한 지표가 된다[9]. 결과지표는 Schumacher과 Meleis [16]가 성공적 이행의 지표로 제시한 주관적 안녕감, 역할 숙달, 관계형성에서의 안녕감을 반영한 삶의 질로 설정하였다. 삶의 질은 한 개인이 살고 있는 문화권과 가치체계의 맥락 안에서 자신의 목표, 기대, 규범, 관심과 관련하여 인생에서 자신이 차지하는 상태에 대한 개인적인 지각이다[10,24]. 중년여성들은 성공적으로 폐경이행을 한 후에는 성취감과 만족감을 느끼는 등 주관적 안녕상태, 즉 삶의 질이 높아진다[1]. 따라서 본 연구는 폐경증상을 폐경이행 속성으로, 회복탄력성과 사회적 지지를 폐경이행을 촉진하고 저해하는 폐경이행 조건으로, 폐경관리, 폐경기 적응, 삶의 질을 폐경이행에 대한 반응패턴으로 폐경이행 설명 모형의 이론적 개념틀을 구축하였다(Figure 1).

이러한 이론적 개념틀을 기반으로 다음과 같은 가설모형을 구축하였다. 폐경증상을 외생변수로, 회복탄력성, 사회적 지지, 폐경기 적응, 폐경관리, 삶의 질을 내생변수로 구성하였다. 변수간의 관계는, 폐경증상은 사회적 지지에, 사회적 지지는 회복탄력성에, 회복탄력성은 폐경관리와 폐경기 적응에, 폐경관리와 폐경기 적응은 삶의 질에 영향을 미치는 경로를 설정하였다(Figure 2). 연구가설은 다음과 같다.

가설 1. 폐경증상은 사회적 지지에 직접적인 효과를 나타낼 것이다.

가설 2. 사회적 지지는 회복탄력성에 직접적인 효과를 나타낼 것이다.

가설 3. 회복탄력성은 폐경기 적응에 직접적인 효과를 나타낼 것이다.

가설 4. 회복탄력성은 폐경관리에 직접적인 효과를 나타낼 것이다.

가설 5. 폐경기 적응은 삶의 질에 직접적인 효과를 나타낼 것이다.

가설 6. 폐경관리는 삶의 질에 직접적인 효과를 나타낼 것이다.

## Methods

Ethics statement: This study was approved by the Institutional Review Board of Chungnam National University (201801-SB-002-01). Informed consent was obtained from the participants.

### 연구 설계

본 연구는 Meleis 등[17]의 이행이론과 문헌고찰을 근거로 폐경기를 경험하는 여성의 폐경이행을 설명하는 가설모형을 제시하고, 선행연구와 수정지수(modification indices)를 검토하여 수정모형을 구축한 후 검증하는 상관성 조사 연구이다.

### 연구 대상

본 연구의 대상자는 폐경이행을 경험하는 여성이다. 대전광역시에 거주하는 만 40–64세의 중년여성 중 폐경증상을 경험하는 기혼 여성을 근접 모집단으로 하였다. 대상자 선정 기준은 주폐경기인 만 40–64세의 기혼이며 Menopause Rating Scale [25]로 평가한 폐경증상 점수가 5점 이상으로 불규칙한 월경을 경험하거나 폐경 후 5년 이내인 여성이다. 대상자 제외기준은 자궁적출술이나 난소절제술로 인공 폐경이 된 자, 여성 호르몬 요법을 받고 있는 자, 만 42세 이전에 조기 폐경된 자이다. 연구자는 연구의 목적을 이해하고 자발적으로 참여에 동의하는 자를 선정 및 제외기준에 따라 임의 표출하였다. 연구자가 직접 대면 모집을 하거나 연구보조원의 도움을 받아 연구 대상자를 모집하였다

최종 대상자는 연구 참여에 동의하고 설문지에 응답한 388명 중 불완전 응답자를 제외한 359명의 여성이다. 이는 최대우도법을 가정한 구조방정식 모형에서 표본 수가 400명 이상일 경우 최대우도법이 민감하게 되어 모델 적합도가 나빠질 수 있고 일반적으로 200이 적당하며, 관측변수당 15배의 표본이 필요하다고 하였으므로[26], 본 연구의 관측변수 21개의 15배인 315명 이상으로 권장 표본크기를 충족하였다.

### 연구 도구

#### 폐경증상

폐경증상은 Heinemann 등[27]의 폐경기 증상이나 이로 인한 불편감을 느낀 정도를 자기 보고식으로 평가하는 Menopause Rating Scale [25]의 한국어판 도구[26]로 측정하였다. 본 도구는 신체적 증상 4문항, 정신적 증상 4문항, 비뇨생식기 증상 3문항의 세 가지 하위척도로 구성된 총 11문항의 5점 Likert 척도이며, ‘전혀 그렇지 않다’ 0점에서 ‘매우 자주 그렇다’ 4점으로 점수 범위는 0–44점이고 점수가 높을수록 폐경증상이 심함을 의미한다. 도구 개발 당시 신뢰도는 Cronbach’s α=.86 [27]이었고, 본 연구의 신뢰도는 .84였다.

#### 회복탄력성

회복탄력성은 Connor와 Davidson [18]이 개발한 탄력성 척도(Connor-Davidson Resilience Scale) 25문항을 Campbell과 Stein이 10문항으로 수정한 도구[28]를 타당화한 한국어판 도구[29]로 측정하였다. 10문항의 5점 Likert 척도로, ‘전혀 그렇지 않다’ 0점에서 ‘매우 그렇다’ 4점으로 점수 범위는 0–40점이고 점수가 높을수록 회복탄력성이 높음을 의미한다. 도구 개발 당시 신뢰도는 Cronbach's α=.85 [28]였고, 본 연구의 신뢰도는 .84였다.

#### 사회적 지지

사회적 지지는 Park [30]이 성인을 대상으로 개발한 사회적 지지 척도 25문항을 Kim [31]이 16문항으로 수정 보완한 도구로 측정하였다. 본 도구는 정서적 지지 4문항, 평가적 지지 5문항, 정보적 지지 3문항, 물질적 지지 4문항의 4개 하부 영역으로 구성되었다. 5점 Likert 척도로 ‘전혀 아니다’ 1점에서 ‘매우 그렇다’ 5점이고, 점수 범위는 16–80점으로 점수가 높을수록 사회적 지지가 높음을 의미한다. 도구 개발 당시 신뢰도는 Cronbach’s α=.94 [31]였고, 본 연구의 신뢰도는 .96이었다.

#### 폐경관리

폐경관리는 Song [32]이 개발한 폐경관리 17문항 척도를 Choi 등[33]이 21문항으로 추가 보완한 도구로 측정하였다. 이 도구는 활동과 운동 관리 3문항, 식생활 관리 3문항, 성생활 관리 4문항, 전문적 건강 관리 3문항, 자가조절 4문항에 식생활 관리 1문항과 자가조절 3문항으로 구성된다. 4점 Likert 척도로 ‘전혀 안 함’ 1점에서 ‘항상 함’ 4점으로 점수 범위는 21–84점이고 점수가 높을수록 폐경관리를 잘 수행하는 것임을 의미한다. 도구 개발 당시 신뢰도는 Cronbach’s α=.82 [33]였고, 본 연구의 신뢰도는 .80이었다.

#### 폐경기 적응

폐경기 적응은 Bae [34]가 개발한 폐경기 적응 측정 도구로 측정하였다. 본 도구는 신체적 변화에 대한 적응 11문항, 자아개념 변화에 대한 적응 4문항, 역할기능 변화에 대한 적응 11문항, 상호의존성 변화에 대한 적응 3문항의 4개 하위영역 총 29문항으로 구성되어 있다. ‘전혀 그렇지 않다’ 5점에서 ‘항상 그렇다’ 1점으로, 22–29번 문항은 역산 처리하고 점수 범위는 29–145점이며 점수가 높을수록 폐경기 적응 수준이 높은 것을 의미한다[35]. 도구 개발 당시 신뢰도는 Cronbach’s α=.88 [34]이었고, 본 연구의 신뢰도는 .86이었다.

#### 삶의 질

삶의 질은 세계보건기구에서 삶의 질 측정도구 World Health Organization Quality of Life Assessment (WHOQOL)-100 [24]를 개발한 후 이를 수정 보완한 간편 척도(WHOQOL instrument, WHOQOL-BREF)를 Min 등[10]이 번안하여 신뢰도와 타당도를 검증한 한국어판 도구로 측정하였다. 본 도구는 신체적 건강 영역, 심리적 영역, 사회적 관계 영역, 환경 영역의 4개 하부 영역 24문항으로 측정하였고, Min 등[10]의 방식에 따라 점수 총합을 계산하였다. 5점 Likert 척도로 ‘매우 나쁨’, ‘매우 불만족’, ‘전혀 아니다’ 1점에서 ‘매우 좋음’, ‘매우 만족’, ‘매우 많이 그렇다’ 5점으로 세 문항(3, 4, 26번)은 역산 처리하였고 점수가 높을수록 삶의 질이 높은 것을 의미한다. 도구 개발 당시 신뢰도는 Cronbach’s α=.65–.93 [24]이었고, 본 연구의 신뢰도는 .92였다.

#### 대상자 일반적 특성

대상자의 연령, 교육수준, 직업 여부, 소득수준, 거주형태, 월경 관련 특성, 건강행위 등을 설문지로 조사하였다.

### 자료수집 방법

자료수집 전에 도구 개발 또는 번역 저자로부터 이메일과 전화를 통해 측정도구에 대한 사용 허락을 받았다. 자료수집 기간은 2018년 4월 1일부터 2018년 6월 30일까지였다. 40–64세 폐경증상이 있는 기혼의 연구대상자에게 연구의 목적과 방법을 설명하고 서면 동의서를 획득한 후 연구참여에 동의한 대상자에게 설문지를 배부하였고 자가작성법을 통해 자료를 수집하였다. 설문 작성시간은 20–40분으로 설문지 작성 후 개인별로 봉투에 밀봉하여 수거하였고, 자발적 참여와 성실한 응답에 대한 보상으로 답례품을 제공하였다. 동의서와 설문지는 별도로 보관하였으며 수집된 자료는 코드화하고 파일은 암호를 지정하여 저장하여 연구 목적 외에는 연구자 외의 타인이 사용할 수 없도록 개인정보를 보호하였다.

### 자료분석 방법

수집된 자료는 IBM SPSS for Windows ver. 24.0과 AMOS ver. 24.0 (IBM Corp., Armonk, NY, USA)을 이용하여 분석하였다. SPSS 프로그램을 이용하여 기술 통계, 차이검정, 상관관계, 신뢰도 분석을 하고, AMOS 프로그램을 이용하여 확인적 요인 분석과 경로 분석을 시행하여 변수의 타당도, 모형적합도, 변수 간 직접효과, 간접효과, 총 효과, 설명력 등 구조방정식모형을 검증하였다.

측정변수 간 다중공선성을 확인하기 위하여 상관계수, 공차(tolerance), 분산팽창계수(variance inflation factor, VIF)로 분석하였다. 구조방정식의 기본 가정인 정규성을 검증하기 위하여 일변량 정규성 검증은 왜도와 첨도로, 다변량 정규성 검증은 다변량 첨도지수로 검토하였다. 다변량 정규성을 가정하는 최대우도법을 이용하여 분석하였다. 확인적 요인 분석은 표준화된 요인 부하량(standardized factor loading; standardized estimates), 임계치의 유의성(critical ration [C.R.]>1.965), 개념신뢰도(construct reliability), 평균분산추출(average variance extracted, AVE)을 통해 타당도 검증을 하였다.

가설모형의 적합도는 절대적합지수인 χ², χ²/degree of freedom (df), goodness of fit index (GFI), standardized root mean square residual (SRMR), root mean square error of approximation (RMSEA), 증분적합지수인 Tucker-Lewis index (TLI), comparative fit index (CFI), incremental fit index (IFI), 간명적합지수인 parsimonious normed fit index (PNFI), Akaike information criterion (AIC) 등을 이용하여 평가하였다. 경로 분석은 경로계수의 유의성을 통해 검증하였고, 부트스트래핑(bootstrapping) (500회)을 적용하여 직접, 간접, 총 효과의 유의성을 확인하였다.

## Results

### 대상자의 일반적 특성

본 연구 대상자는 40–61세로 평균 연령은 47.80세였다. 교육수준은 대학교 졸업이 231명(64.3%)이었고, 거주형태는 308명(85.8%)이 가족과 함께 거주하였다. 경제상태는 286명(79.7%)이 중간으로, 직업이 있는 경우는 204명(56.8%)으로 응답했다. 월경 상태는 규칙적인 상태가 152명(42.3%), 불규칙적인 상태가 140명(39.0%), 폐경 후 상태가 67명(18.7%)이었으며 폐경 연령은 46–59세로 평균 52.03세였다. 대상자의 일반적 특성에 따른 삶의 질 차이검정 결과, 학력과 수입에 따라 삶의 질에 유의한 차이가 있었다. 즉 중학교 졸업 이하 학력을 가진 여성의 삶의 질이 고등학교, 대학교, 대학원 졸업 학력을 가진 여성의 점수보다 낮았다(F=8.56, *p*<.001). 또한 경제상태가 높은 군에 비해 중간 및 낮은 수준인 군에서 삶의 질이 낮았다(F=12.52, *p*<.001) (Table 1).

### 연구 변수의 기술 통계와 정규성 및 다중공선성 평가

폐경증상은 최고 35점 점수 범위에서 평균 12.88점이었다. 회복탄력성은 최고 40점의 점수 범위에서 평균 26.04점이었고, 사회적 지지는 최고 80점 범위에서 평균 61.33점이었다. 폐경기 적응은 최고 123점 범위에서 평균 96.35점이었고 폐경관리는 최고 79점 범위에서 평균 50.97점이었다. 삶의 질은 최고 129점 범위에서 평균 90.60점이었다(Table 2). 폐경증상은 폐경관리 외에 다른 변수들 간에는 유의미한 음의 상관관계를(r=–.50 to –.25, *p*<.001), 회복탄력성, 사회적 지지, 폐경기 적응, 폐경관리, 삶의 질 간에는 유의미한 양의 상관관계를 나타내었다(r=.22–.58, *p*<.001).

연구변수들의 일변량 정규성을 검토한 결과 왜도는 –0.55에서 0.84, 첨도는 –0.47에서 0.94로, .05 유의수준에서 왜도는 ±1.96 이하, 첨도는 절대값이 7 이하이면 정규분포 가정을 만족하므로[26] 일변량 정규성 가정은 만족하였다. 다변량 첨도 지수는 66.66으로 정규성의 가정을 위배하는 것으로 나타났다. 본 연구에서는 모수 추정을 위해 최대우도법을 기본 추정법으로 사용하였는데, 이는 다변량 정규분포를 하지 않더라도 데이터의 분포가 정규분포에 근접하고 5점 이상 Likert 척도를 사용한 경우 최대우도법을 사용할 수 있다. 또한 부트스트래핑은 다변량 정규분포의 가정으로부터 자유롭기 때문에 다변량 정규성을 벗어난 데이터 분석에 유용하게 사용된다[26]는 견해에 따라 분석을 수행하였다.

오차의 자기상관 검증을 위한 Durbin-Watson 값은 1.89로 2에 가까운 값이므로 자기상관이 없는 것으로 확인되었다. 잠재변수들 간의 상관관계 계수의 절대값은 .59 이하, 공차는 .54에서 .99, VIF는 1.02에서 1.86로 나타나, 상관관계 계수의 절대값 .80 미만[20], 공차 0.1 이상, VIF 10 미만의 기준을 충족하여 다중공선성은 없는 것으로 판단하였다.

### 폐경이행 설명 모형의 검증

#### 확인적 요인 분석

확인적 요인 분석을 통해 집중타당성과 판별타당성을 통한 측정모형의 타당도를 검증하였다. 우선 집중타당성은 표준화된 요인부하량과 임계치의 유의성, AVE와 개념신뢰도로 확인하였다. 요인부하량은 .50에서 .95, 유의성은 C.R.=1.965 이상, 개념신뢰도는 .70 이상, AVE는 .50 이상일 때의 기준에 따라 집중타당성을 평가[26]한 결과, 모든 연구변수는 집중타당성을 갖고 있었다. 판별타당성은 가장 높은 변수 간 상관을 택하여 ‘AVE는 상관계수의 제곱 값보다 크다’의 기준에 따라 판별타당성을 평가[26]한 결과, 본 연구의 변수도 판별타당성을 갖고 있음을 확인하였다(Table 2).

#### 가설적 모형의 검정 및 수정

가설적 모형의 적합도를 검증한 결과 절대적합지수인 *χ*^2^=724.29, *χ*^2^/df=3.94이었고 GFI=.84, RMSEA=.09, SRMR=.12이었다. 증분적합지수인 CFI=.85이고 TLI=.82, IFI=.85이었으며, 간명적합지수인 PNFI=.71, AIC=818.29로 적합도 지수는 권장기준에 충족되지 않았다. 이에 모형의 적합도를 높이기 위해 수정지수를 참조하여 사회적 지지가 폐경기 적응에 영향을 미치는 것[22]과 변수 간 관계에 대한 경험적 근거를 탐색하여 폐경증상이 폐경기 적응에 대해 영향을 미치는 것[35]을 바탕으로 가설모형에 2개의 경로를 추가하고, 오차 간의 상관관계를 설정하는 등 수정모형을 설정하였다. 우선 수정지수 35.934, parameter change (Par Change) 0.157과 사회적 지지가 폐경기 적응에 영향을 미치는[22] 선행연구를 근거로 폐경증상에서 사회적 지지로의 경로를 추가하였다. *χ*^2^=653.93, *χ*^2^/df=3.57, GFI=.85이고 RMSEA=.09, 증분적합지수인 CFI=.87, TLI=.85, IFI=.87이었으며 간명적합지수인 PNFI=.72, AIC=749.93이었다. 폐경증상에서 폐경기 적응으로의 이론적 근거[35]에 의한 경로를 추가하였다. *χ*^2^=605.33, *χ*^2^/df=3.33, GFI=.85이고 RMSEA=.08, 증분적합지수인 CFI=.88, TLI=.86, IFI=.88이었으며 간명적합지수인 PNFI=.73, AIC=703.33이었다. 수정지수 26.26, Par Change 0.042를 근거로 오차인 e7과 e8를 연결하는 경로를 추가하였다. *χ*^2^=574.48, *χ*^2^/df=3.17, GFI=.86이고 RMSEA=.08, 증분적합지수인 CFI=.89, TLI=.87, IFI=.89이었으며 간명적합지수인 PNFI=.73, AIC=674.48이었다. 수정지수 18.87, Par Change –0.077을 근거로 오차인 e14와 e17을 연결하는 경로를 추가하였다. *χ*^2^=552.97, *χ*^2^/df=3.07, GFI=.87이고 RMSEA=.08, 증분적합지수인 CFI=.89, TLI=.88, IFI=.89였으며 간명적합지수인 PNFI=.73, AIC=654.97이었다. 수정지수 11.73, Par Change 0.037을 근거로 오차인 e15와 e16을 연결하는 경로를 추가하였다 *χ*^2^=538.92, *χ*^2^/df=3.01, GFI=.87이고 RMSEA=.08, 증분적합지수인 CFI=.90, TLI=.88, IFI=.90이었으며 간명적합지수인 PNFI=.73, AIC=642.92였다. 최종적으로 수정지수 7.45, Par Change 0.029를 근거로 오차인 e10과 e12를 연결하는 경로를 추가하였다 최종 수정모형의 적합도는 절대적합지수 *χ*^2^=522.26, *χ*^2^/df=2.93, GFI=.88, RMSEA=.07, SRMR=.07, 증분적합지수 CFI=.90, TLI=.88, IFI=.90, 간명적합지수 PNFI=.73, AIC=628.2를 보여, 가설모형에 비해 향상되었다.

#### 수정모형의 경로 분석 및 효과 검증

**Figure 3.**
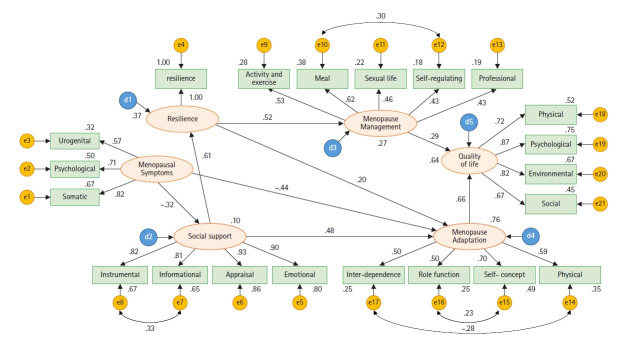
Modified hypothetical model.

수정모형에서는 8개의 경로가 모두 유의하여 직접효과를 지지하였다(Figure 3). 수정지수를 통한 수정모형의 가설에 대한 결과는 다음과 같다.

가설 1. 폐경증상은 사회적 지지에 직접적인 효과를 나타낼 것이다. 심한 폐경증상은 사회적 지지에 대하여 β=–.32로 부정적인 직접효과를 나타내므로 지지되었다.

가설 2. 사회적 지지는 회복탄력성에 직접적인 효과를 나타낼 것이다. 사회적 지지는 회복탄력성에 대하여 β=.61로 긍정적인 직접효과를 나타내므로 지지되었다.

가설 3. 회복탄력성은 폐경기 적응에 직접적인 효과를 나타낼 것이다. 회복탄력성은 폐경기 적응에 대해 β=.20으로 긍정적인 직접효과를 나타내므로 지지되었다.

가설 4. 회복탄력성은 폐경관리에 직접적인 효과를 나타낼 것이다. 회복탄력성은 폐경관리에 대해 β=.52로 긍정적인 직접효과를 나타내므로 지지되었다.

가설 5. 폐경기적응은 삶의 질에 직접적인 효과를 나타낼 것이다. 폐경기 적응은 삶의 질에 대해 β=.66으로 긍정적인 직접효과를 나타내므로 지지되었다.

가설 6. 폐경관리는 삶의 질에 직접적인 효과를 나타낼 것이다. 폐경관리는 삶의 질에 대해 β=.30으로 긍정적인 직접효과를 나타내므로 지지되었다. 수정된 가설인 심한 폐경증상이 폐경기 적응에 대해 β=–.44로 부정적인 직접효과를 나타내므로 지지되었다. 사회적 지지가 폐경기 적응에 대해 β=.48로 긍정적인 직접효과를 나타내므로 지지되었다.

또한 수정모형의 내생변수에 대한 설명력은 삶의 질에 대해 63.6%, 폐경관리의 경우 27.5%, 폐경기 적응의 경우 76.0%였다.

간접효과를 나타낸 경로를 살펴보면, 심한 폐경증상은 사회적 지지를 통하여 회복탄력성(β=–.19), 폐경기 적응(β=–.19)에, 회복탄력성을 통해 폐경기 관리(β=–.10)에, 폐경기 적응과 관리를 통해 삶의 질 (β=–.45)에 부정적인 효과를 보였다. 반면 사회적 지지는 회복탄력성을 통하여 폐경기 적응(β=.12) 및 폐경관리(β=.32)에, 폐경기 적응 및 폐경관리를 통해 삶의 질(β=.49)에 긍정적 효과를 보였다. 또한 회복탄력성은 폐경기 적응과 폐경관리를 통하여 삶의 질(β=.29)에 긍정적 효과를 보였다(Table 3)

#### 통제변수가 포함된 수정모형의 분석 결과

대상자의 삶의 질에 유의한 차이를 보인 교육수준과 경제상태를 통제변수로 포함하여 수정모형을 분석한 결과, 이행반응 패턴에 대해 수정모형 경로에 유의한 차이가 나타나지 않았다. 즉 수정모형에 대한 통제변수의 영향력은 나타나지 않았다.

## Discussion

본 연구는 이행이론을 기반으로 폐경이행을 설명하기 위해 문헌고찰에 따라 개념과 문장을 도출하여 이론적 기틀에 따른 가설모형을 구축하였고, 가설모형과 수정한 모형을 검증하였다. 수정모형 검증 결과 폐경증상이 사회적 지지와 회복탄력성을 통해 폐경관리와 폐경기 적응을 통과하여 삶의 질에 도달하게 되는 과정을 확인하였기에 이에 대해 논의하고자 한다.

본 연구의 이행이론을 근거로 한 가설모형은 집중타당성과 판별타당성을 통해 연구변수의 타당도를 검증하였다. 모델적합도를 측정한 결과 본 연구의 가설모형은 모델적합도 지수가 권장기준을 충족하지 못하였다. 이에 수정지수에 근거하여 사회적 지지가 폐경기 적응을 촉진하고, 폐경증상을 심하게 호소할수록 폐경기 적응 수준은 낮다[2]는 선행연구에 근거하여 폐경기 적응에 각각 직접 경로를 추가한 결과, 수정모형의 적합도가 향상되었다. 이행이론을 기반으로 부분적인 폐경이행 조건이 자아정체감과 삶의 질로 설정된 폐경이행 반응패턴에 직접 효과를 나타냄을 검증한 Hong과 Kang [1]의 연구와 달리, 본 연구에서는 이행조건, 반응패턴까지 통과하는 이행이론의 틀을 유도한 설명모형을 검정하여 이론의 유용성을 확인하였다.

수정모형의 경로에 대해 모두 유의한 직접, 간접 효과를 검증하여 폐경이행 과정의 경로 설정이 적절함을 확인하였다. 회복탄력성과 사회적 지지가 폐경기 적응과 폐경관리에 유의한 영향을 미친 것은 폐경이행의 조건이 이행과정에서 촉진 효과를 지지한 것이다. 폐경기 적응과 폐경관리가 삶의 질에 영향을 미친 것은 폐경관리가 삶의 질과 자아정체감으로 측정된 폐경이행의 영향요인[1]이고 폐경기 적응의 하부요인이 삶의 질에 영향을 미친 연구[9]와 유사하다. 따라서 폐경이행을 설명하기 위한 본 연구의 폐경이행 설명모형은 모델 구성이 적합하고 폐경증상, 회복탄력성, 사회적 지지, 폐경관리, 폐경기 적응, 삶의 질의 관련성에 대한 경로 설정이 타당하다고 여겨진다. 이행이론을 통한 폐경이행에 대한 설명모형은 과정지표인 폐경관리에서 27.5%, 폐경기 적응에서 76.0%를, 결과지표인 삶의 질에 대해 63.6%의 설명력을 보여 이행이론에 근거한 폐경이행 설명모형이 적절함을 확신할 수 있다.

폐경이행 조건인 회복탄력성과 사회적 지지는 폐경관리와 폐경기 적응을 통해 삶의 질에 영향을 주었다. 이는 회복탄력성의 긍정성의 속성을 볼 때, 자신의 삶에 대해 긍정적으로 생각하고 능동적으로 관리하는 것이 폐경기 여성으로 하여금 스스로 폐경기에 잘 적응할 수 있게 한다[35]는 견해에 의해 지지되었다. 사회적 지지는 또 다른 폐경이행의 촉진요인으로, 회복탄력성에 직접 영향을 미치고 폐경기 적응과 삶의 질에 직•간접적인 영향을 미쳤다. 선행연구에서도 부인암 환자의 가족 지지가[19] 회복탄력성에 영향을 미치는 강력한 요인으로 보고되었다. 또한 사회적 지지는 개인의 위기, 스트레스 상황에서 지지체계들의 기능 여부에 의해 문제 상황을 극복하고 적응하는 외적 요인으로 보고되었기에[36] 폐경기 적응을 직접적으로, 내적 요인인 회복탄력성을 통해[19,37] 폐경기 적응, 폐경관리를 간접적으로 촉진하여 삶의 질을 증진한다고 볼 수 있다. 심한 폐경증상이 폐경관리, 폐경기 적응 및 삶의 질에 부정적인 영향을 미치는 관계에서 높은 회복탄력성과 사회적 지지가 폐경관리와 폐경기 적응을 증진하는 직∙간접적인 촉진요인임을 확인하였다. 따라서 폐경이행기 여성의 폐경이행 과정을 탐색할 때 이들의 폐경이행 조건에 대한 간호사정을 수행할 필요가 있다. 또한 선행연구에서[1] 폐경증상과 자아 분화가 폐경관리에 대해 9.6%의 낮은 설명력을 보인 반면, 본 연구의 회복탄력성과 사회적 지지는 폐경이행에 대한 촉진요인으로서 폐경관리에 대해 27.5%의 설명력을 보여 폐경이행 조건의 촉진요인으로 확인되었다. 따라서 회복탄력성과 사회적 지지를 폐경이행기 여성의 삶의 질을 증진하는 간호중재 시 중요한 지표로 고려해야 할 것이다.

결론적으로 가설모형 구축과 검증을 통해 폐경이행기 여성들은 폐경증상이 심할수록 폐경기 이후 삶의 질은 부정적인 상태 즉, 삶의 질이 저하된 상태로 살아간다고 하나, 이행조건인 사회적 지지와 회복탄력성을 통해 긍정적인 폐경관리와 폐경기 적응의 과정을 거쳐 삶의 질을 유지하거나 증진할 수 있는 폐경이행을 통과하게 된다는 것을 확인하였다. 연구결과에 따라 간호사는 폐경증상을 경험하는 여성에게 폐경이행에서 촉진요인인 스트레스를 받을 때도 집중력과 사고력을 잘 유지하며 강한 자신을 믿고 재미있는 면을 찾으려고 하는 긍정성을 증강하는 회복탄력성 강화 프로그램 또는, 이해하고 관심 갖고 사랑받고 있다는 느낌을 갖게 하고 정보와 충고, 도움과 시간을 제공하는 사회적 지지체계를 강화할 수 있는 간호중재 프로그램의 개발 전략을 세워야 한다.

본 연구의 대상자는 일부 지역 기혼 여성의 편의 모집으로 인해 고학력과 경제상태가 중간 이상인 특성을 갖고 있기 때문에, 본 연구 결과를 일반인에게 확대 해석하는 데 주의를 요한다. 또한 본 연구는 설명모형을 구축하고 횡단적 자료를 구조방정식 모형 검정을 통해 인과관계로 해석하였기에 해석에 주의가 필요하다. 추후 연구에서는 폐경이행 현상을 시간적 경과에 따른 속성, 조건, 반응패턴을 통해 설명할 수 있기에 종단적 연구를 통해 폐경이행 과정을 이해할 필요가 있다. 폐경증상을 경험하는 대상자의 삶의 질 증진을 위해 이행조건의 역할을 중심으로 폐경관리와 폐경기 적응을 강화할 내적•외적인 촉진 및 저해요인을 탐색할 필요가 있다.

## Figures and Tables

**Table 1.** Characteristics of the participants (N=359)

Characteristic	Categories	n (%)	Mean±SD (range)	Quality of life	
				Mean±SD	t or F^†^(*p*)
Age (year)			47.80±5.11 (40–61)		
Level of education	≤Middle school^a^	7 (1.9)		70.14±16.92	8.56 (<.001)
	High school^b^	57 (15.9)		87.51±12.47	a<b, c, d
	College^c^	231 (64.3)		91.29±12.37	
	≥Graduate school^d^	64 (17.8)		93.11±12.30	
Living arrangement	As a couple	51 (14.2)		91.41±15.67	0.49 (*.*628)
	With multiple family members	308 (85.8)		90.47±12.35	
Economic status	Low^a^	39 (10.9)		82.69±13.21	12.52 (<.001)
	Middle^b^	286 (79.6)		90.91±12.53	a<b<c
	High^c^	34 (9.5)		97.06±10.85	
Occupation	No	155 (43.2)		89.31±12.67	–1.67 (.097)
	Yes	204 (56.8)		91.58±12.93	
Menstruation	Regular	152 (42.3)		91.44±11.78	1.02 (.361)
	Irregular	140 (39.0)		90.58±13.51	
	Menopause	67 (18.7)	52.03±3.00^‡^ (46–59)	88.75±13.73	

† Bonferroni correction.

‡ Age at menopause.

**Table 2.** Descriptive statistics and confirmatory factor analysis for measured variables

Variable	Mean±SD	Standardized estimate	CR	AVE	r for correlation					
					MS	Re	SS	MA	MM	QoL
Menopausal symptoms (MS)	12.88±6.42		0.84	0.65						
Somatic	4.62±2.83	0.81								
Psychological	5.17±2.74	0.71								
Urogenital	3.09±2.34	0.57								
Resilience (Re)	26.04±5.96				–.25					
Social support (SS)	61.33±9.52		0.97	0.88	–.27	.58				
Emotional	15.45±2.48	0.90								
Evaluation	19.30±3.29	0.91								
Information	11.28±2.06	0.83								
Instrumental	15.30±2.65	0.84								
Menopause adaptation (MA)	96.35±11.52		0.85	0.59	–.50	.48	.48			
Physical	34.19±5.94	0.59								
Self concept	14.57±3.02	0.78								
Role function	36.85±4.58	0.55								
Inter-dependence	10.73±2.40	0.45								
Menopause management (MM)	50.97±7.97		0.83	0.51	.01	.39	.31	.22		
Activity and exercise	6.97±2.24	0.46			(.791)					
Meal	10.54±2.39	0.73								
Sexual life	7.94±2.19	0.43								
Self-regulating	19.72±3.34	0.56								
Professional	5.80±2.10	0.42								
Quality of life (QoL)	90.60±12.85		0.95	0.81	–.33	.54	.57	.51	.33	
Physical	24.70±4.37	0.71								
Psychological	20.62±3.35	0.87								
Environment	27.57±4.74	0.82								
Social	10.67±1.70	0.67								

AVE: Average variance extracted; CR: construct reliability

**Table 3.** Standardized direct, indirect, and total effects in the modified theoretical model

Endogenous variable	Exogenous variable	SMC	Standardized direct effect, β (CI)	Standardized indirect effect, β (CI)	Standardized total effect, β
Social support	Menopausal symptoms	.10	–.32 (–0.44 to –0.19)		–.32
Resilience	Menopausal symptoms	.37		–.19 (–0.27 to –0.11)	–.19
	Social support		.61 (0.53–0.6*8*)		.61
Menopause adaptation	Menopausal symptoms	.76	–.44 (–0.60 to –0.30)	–.19 (–0.27 to –0.12)	–.63
	Social support		.48 (0.34–0.61)	.12 (0.06–0.19)	.60
	Resilience		.20 (0.10–0.32)		.20
Menopause management	Menopausal symptoms	.28		–.10 (–0.15 to –0.06)	–.10
	Social support			.32 (0.23–0.41)	.32
	Resilience		.52 (0.40–0.65)		.52
Quality of life	Menopausal symptoms	.64		–.45 (–0.54 to –0.34)	–.45
	Social support			.49 (0.39–0.60)	.49
	Resilience			.29 (0.20–0.39)	.29
	Menopause adaptation		.66 (0.54–0.77)		.66
	Menopause management		.30 (0.14–0.44)		.30

SMC: Squared multiple correlations.

## References

[1] Hong E, Kang YS (2015). Structural equation modeling on healthy menopausal transition. J Korean Acad Nurs.

[2] Lee EJ (2018). Factors influencing adaptation to menopause in middle-aged women. Korean J Women Health Nurs.

[3] Kim JH, Kim MR, Lee YJ, Hwang SJ, Jo HH, Ryu KS (2003). Serum levels and expressions of Inhibin A and Inhibin B in the ovaries of perimenopausal women. Korean J Obstet Gynecol.

[4] Lee JH, Kim KH, Kim GD (2014). The mediating effect of cognitive function on climacteric symptoms and quality of life in the middle-aged women. J Korea Acad-Ind Coop Soc.

[5] Yang KM (2015). The effect of depression, life stress and resilience on quality of life in middle aged women. J Korean Acad Soc Home Care Nurs.

[6] Kim KH (2016). The factors influencing to quality of life of middle-aged women. J Korean Data Anal Soc.

[7] Yeom YR, Kim A (2021). Structural equation modeling on quality of life in middle-aged women with urinary incontinence. J Korean Acad Fundam Nurs.

[8] Kim AK (2010). Yangsaeng and health related quality of life (HRQOL) in middle aged women. Korean J Women Health Nurs.

[9] Kim MJ, Kang KJ (2014). Effects of sanhujori and menopausal adaptation on health-related QOL in middle-aged women. Korean J Women Health Nurs.

[10] Min SK, Lee CI, Kim KI, Suh SY, Kim DK (2000). Development of Korean version of WHO quality of life scale abbreviated version (WHOQOL-BREF). J Korean Neuropsychiatr Assoc.

[11] Lee YM, Kim GM, Jung YH (2014). Factors affecting a health promoting lifestyle in middle-aged women. J Korea Content Assoc.

[12] Song AR (2001). An analysis of the relationship between climacteric symptoms and management of menopause in middle-aged women. J Korean Acad Soc Nurs Educ.

[13] Kim KW, Bae KE (2017). Crisis adaptation of middle-aged women during climacteric period. Crisisonomy.

[14] Yoon JH, Han JH (2013). A study on the psychological phenomenon experienced by menopausal middle-aged women. Korean J Couns.

[15] Kim AJ (2008). QOL-BREF and Yangsaeng in Korean adult. J East-West Nurs Res.

[16] Schumacher KL, Meleis AI (1994). Transitions: a central concept in nursing. Image J Nurs Sch.

[17] Meleis AI, Sawyer LM, Im EO, Hilfinger Messias DK, Schumacher K (2000). Experiencing transitions: an emerging middle-range theory. ANS Adv Nurs Sci.

[18] Connor KM, Davidson JR (2003). Development of a new resilience scale: the Connor-Davidson Resilience Scale (CD-RISC). Depress Anxiety.

[19] Cho HM, Yoo EK (2015). Effects of depression, family support on resilience in patients with gynecological cancer. J Korea Soc Wellness.

[20] Yang JH, Kim OS (2016). The structural equation model on resilience of breast cancer patients receiving chemotherapy. J Korean Acad Nurs.

[21] Chang KM (2011). Moderating effects of psychological resilience of the survivors of breast cancer patients on the influential relationships among the variables of body change stress, depression, and social adjustment. Korean J Stress Res.

[22] Kim YS, Chung HJ (2014). The effect of family support and self-differentiation on the college life adjustment of midlife married women students. Korean J Family Welf.

[23] Im EO, Meleis AI (1999). A situation-specific theory of Korean immigrant women’s menopausal transition. Image J Nurs Sch.

[24] The World Health Organization Quality of Life Assessment (WHOQOL) (1998). development and general psychometric properties. Soc Sci Med.

[25] ZEG Berlin (c2008). MRS-the Menopause Rating Scale developed by the Berlin Center for Epidemiology and Health Research [Internet]. https://zeg-berlin.de/expertise/diagnostics-tools/menopause-rating-scale/languages/.

[26] Yu JP (2012). The concept and understanding of structural equation modeling.

[27] Heinemann K, Ruebig A, Potthoff P (2004). The Menopause Rating Scale (MRS) scale: a methodological review. Health Qual Life Outcomes.

[28] Campbell-Sills L, Stein MB (2007). Psychometric analysis and refinement of the Connor-davidson Resilience Scale (CD-RISC): validation of a 10-item measure of resilience. J Trauma Stress.

[29] Jung SY, Nam IS, You S (2016). Validity and factor structure of the Connor-Davidson resilience scale in older adults in Korea. J Korea Gerontol Soc.

[30] Park JW (1985). A study to development a scale of social support. [dissertation].

[31] Kim YS (2013). The relationships among social support, self-esteem, depression and happiness of the retired leisure male sports participants. [dissertation].

[32] Song AR (1998). Development of an educational program for the management of menopause and it’s effect. J Korean Acad Nurs.

[33] Choi NY, Choi SY, Jo HJ (1998). A study on the degree of knowledge of menopause and management of menopausal women. Korean J Women Health Nurs.

[34] Bae KE (2006). Instrument development for adaptation of women in the menopause period. [dissertation].

[35] Park HS, Kim AJ, Bae KE (2010). Life stress, life satisfaction, and adaptation of middle-aged women in the menopause period. Korean Parent-Child Health J.

[36] So IS, Jeong HS (2017). Predictive factors to health promotion behaviors in breast cancer patients using Pender’s health promotion model. J Korea Acad-Ind Coop Soc.

[37] Kim YW, Choi HK, Oh EJ (2018). Convergence study of menopausal resilience of middle-aged women. J Digit Converg.

